# In-Situ Bubble Stretching Assisted Melt Extrusion for the Preparation of HDPE/UHMWPE/CF Composites

**DOI:** 10.3390/polym11122054

**Published:** 2019-12-11

**Authors:** Xiaochun Yin, Youhua Yin, Di Cheng, Yanhong Feng, Guizhen Zhang, Jinsong Wen

**Affiliations:** 1 Key Laboratory of Polymer Processing Engineering of Ministry of Education, South China University of Technology, Guangzhou 510640, China; xcyin@scut.edu.cn (X.Y.); scutyyh@163.com (Y.Y.); scutcd@163.com (D.C.); jswen@scut.edu.cn (J.W.); 2 Guangdong Provincial Key Laboratory of Technique and Equipment for Macromolecular Advanced Manufacturing, South China University of Technology Guangzhou, Guangzhou 510640, China

**Keywords:** ultra-high molecule weight polyethylene (UHMWPE), carbon fiber (CF), high density polyethylene (HDPE), extensional deformation, in-situ bubble stretch (ISBS)

## Abstract

In this work, a novel melt extrusion method under synergy of extensional deformation and in-situ bubble stretching (ISBS) and corresponding apparatus were reported. The structure and working principle were introduced in detail. Polymer composites composed of high density polyethylene (HDPE)/ultrahigh molecular weight polyethylene (UHMWPE)/carbon fiber (CF) were prepared by using this new method. Effects of CF and Azodicarbonamide (AC) contents on composites’ morphology, rheological, thermal, and mechanical properties were experimentally investigated. SEM results showed that the CFs dispersed evenly in the matrix when the AC content was relatively high. DSC results showed that co-crystallization of HDPE and UHMWPE occurred in the composites, and the *X*c of the composites decreased with the addition of AC or under high CF loadings. TGA results showed that the thermostability of the composites increased markedly with increasing CF loading. Mechanical properties showed that tensile strength increased by 30% with 9 wt % CF and 0.6 wt % AC added. The results aforementioned indicate that the novel melt extrusion method is a green and effective way to prepare HDPE/UHMWPE/CF composites.

## 1. Introduction

High density polyethylene (HDPE) has been widely used as a potential structural matrix for various industrial applications due to its good mechanical properties and ease in fabrication [1]. However, compared with engineering polymers, its strength is relatively low. In order to reinforce its properties, the simple and effective way is to make polymer blends with other polymer materials such as PA6 [2], PP [3] and ultra-high molecular weight polyethylene UHMWPE [4], inorganic fillers [5,6,7,8], or organic fillers [9,10,11] to achieve complementary advantages between different materials.

To date, the modification of HDPE with UHMWPE has gained great interest among researchers [12,13,14], because the compounding of such polyethylene with similar chemical nature may lead to synergistic effect in terms of enhancement in impact toughness and other mechanical properties. UHMWPE is capable to confer many excellent properties on HDPE materials, such as low friction and wear rate, as well as excellent fatigue resistance. Meanwhile, HDPE/UHMWPE blends have better creep resistance than neat UHMWPE [15]. Yet, the properties of HDPE/UHMWPE blends have much room for further improvement due to insufficient chain entanglement across the phase interfaces between HDPE and UHMWPE, which is caused by extremely slow chain diffusion of UHMWPE [16]. It has been reported that the chain diffusion coefficient increases with increasing temperature in previous research [17]. However, the increased temperature may accelerate degradation of molecular chain, which lead to a decrease in product properties. Introducing carbon fiber (CF) into the HDPE/UHMWPE blend is another possible method to enhance the comprehensive properties. 

As a result of the characteristic of extremely high strength and modulus, good stiffness, creep resistance, and low density, CF has been widely employed as an ideal reinforcement for advanced composite [18]. CF-reinforced composites are increasingly utilized for a variety of high-performance applications such as the aerospace industry and automobile industry owing to their excellent specific mechanical properties [19]. However, the properties of CF-reinforced composites depend on the dispersion of CF which is determined by the processing equipment and method.

Melt blending is suitable for industrialization due to its well-designed processing equipment and simple process [20]. Most of the equipment for melt blending such as internal mixer [21], twin-screw extruder [22] are dominated by shear deformation. However, there still exist some deficiencies like incomplete dispersion and distribution of primary CF agglomerates or the insufficient adhesion between CFs and the polymer matrix. It is testified that the dispersing efficiency under the extensional deformation is twice that under shear deformation when the deformation rate is equal [23,24]. To generate elongation flow and enhance the mixing efficiency, some novel mixing methods and corresponding devices such as vane extruder (VE) [25,26], vane mixer (VM) [27] were proposed. Zou [28] investigated the influence of processing temperature and rotor speed on dispersive mixing performance and mechanical properties of PP/EPDM blends in a VE. Huang [29] studied the effects of mixing time and rotor speed on dispersion of MMT in HDPE matrix by using a vane mixer. The results showed that commercial MMT could be exfoliated and intercalated in HDPE/MMT nanocomposites without any additive, which indicated a great mixing efficiency. In addition, it is an effective way to improve the mixing efficiency and performance by superimposing external force field onto elongation flow or shear flow. Yin [30] designed a novel mixing device in which ultrasonic vibration synergized with extensional deformation acts on the melts. The morphology results showed that CNTs dispersed homogeneously in intractable UHMWPE matrix without any aid of additives or solvents. Wu [31] proposed a method named in-situ bubble stretching to promote the dispersion of fillers in matrix and improve the composites’ properties without modifying the structure of the mixing device. This method has been used to prepare polymer-based composites such as HDPE/Nano-CaCO_3_ [31], GFO/pPI [32], LDPE/MWCNTs [33], HDPE/nano-SiC [34], which verified that in-situ bubble stretching (ISBS) is an efficient and environmentally friendly method to realize uniform dispersion of the fillers. Furthermore, the feasibility of the synergy of ISBS and extensional deformation was also confirmed according to the research by Yang [33] and Yin [34].

To our knowledge, there has been no work done to enhance the dispersion of CF in polymer matrix under the synergy of ISBS and extensional deformation by now. In this research, melt extrusion process under synergy of in-situ bubble stretching and extensional deformation was developed and the compounding of the HDPE/UHMWPE/CF composites was carried out. The effects of CF and Azodicarbonamide (AC) contents on morphological, rheological, thermal, and mechanical properties of the HDPE/UHMWPE/CF composites were experimentally discussed.

## 2. Experimental

### 2.1. Vane Extruder

**Figure 1:**
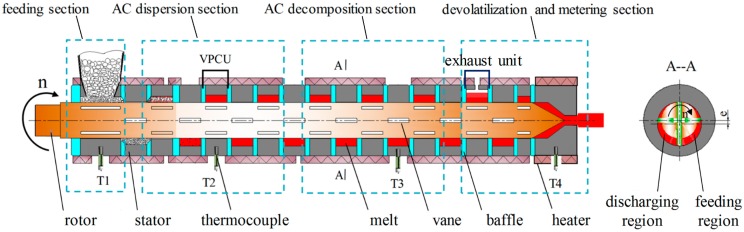
Schematic structure of vane extruder dominated by extensional deformation.

In this research, a novel equipment named vane extruder (VE) dominated by extensional deformation was used to prepare HDPE/UHMWPE/CF composites. As shown in Figure 1, the vane extruder was divided into feeding, AC dispersion, AC decomposition, devolatilization, and metering section according to the entire compounding process in this study. The temperature profile from feeding section to metering section was *T*_1_ = 170 °C, *T*_2_ = 175 °C, *T*_3_ = 210 °C, and *T*_4_ = 200 °C, respectively. The temperature in the AC dispersion section was below the decomposition temperature of the AC. Each section contains several vane plasticizing and conveying units (VPCU). In a VPCU, the stator, rotor, vanes, and baffles make up the chamber with a cross section shape of an eccentric circular ring. The volume of the chamber changes from big to small or small to big periodically as the rotor rotating, which is attributed to a certain eccentricity between the centers of stator and rotor. The region where the volume decreases is called discharging region. On the contrary, the region where the volume increases is called feeding region (as shown in Figure 1A--A). When the volume of the chamber becomes large, polymer materials are fed into it, and then ground and compacted. After going through all the VPCUs in the VE, the polymer materials are eventually compounded and then discharged from the outlet, completing a plasticizing and positive displacement conveying process based on elongation rheology in a short thermo-mechanical history [35], which will reduce the degradation of polymer during processing. The structure and working principle were introduced in detail in our previous researches [26,27,36].

### 2.2. Compounding Process under Synergy of Extensional Deformation and ISBS

**Figure 2:**
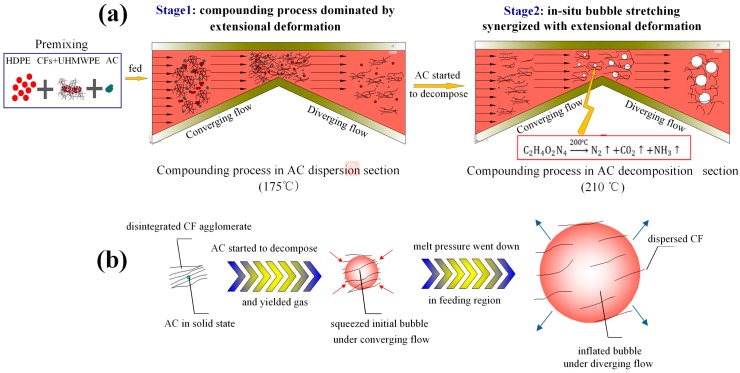
(**a**) Schematic of compounding process under synergy of extensional deformation and in-situ bubble stretching (ISBS). (**b**) Diagram for the mechanism of ISBS’ dispersion effect on agglomerated carbon fibers (CFs).

The schematic of compounding process under synergy of extensional deformation and ISBS was presented in Figure 2. As depicted in Figure 2a, there were two stages in the compounding process: compounding process dominated by extensional deformation (stage 1) and compounding process under synergy of extensional deformation and ISBS (stage 2). The entire compounding process can be described as follows:

#### 2.2.1. Feeding

HDPE, UHMWPE, CFs, and AC were premixed and fed into the feeding section. The UHMWPE powder effectively inhibited the CFs agglomeration caused by electrostatic adsorption. As the rotor rotated, materials were positive displacement conveyed and forced out into the AC dispersion section after being gradually squeezed. 

#### 2.2.2. Compounding Dominated by Extensional Deformation

In the AC dispersion section, pulsatile extensional deformation generated by periodically changed converging/diverging flow was acted on the materials when the materials flowed from discharging region to feeding region repeatedly between different VPCUs. AC, CFs, and UHMWPE gradually dispersed in HDPE resin. Meanwhile, AC dispersed in CF agglomerates and among the phases of UHMWPE and HDPE (Figure 2a: stage 1). AC, CFs, and UHMWPE had primarily dispersed in HDPE resin before the materials flowed into the AC decomposition section. The AC dispersed in the CFs agglomerates, the phase interface and in the HDPE resin respectively was in solid state as the melt temperature was under the decomposition temperature of the AC.

#### 2.2.3. Compounding under Synergy of Extensional Deformation and ISBS

As shown in Figure 2a: stage 2, after the materials were forced to the AC decomposition section, the AC distributing among disintegrated CF agglomerates and between the phases of UHMWPE and HDPE decomposed as the melt temperature in the AC decomposition section is above the decomposition temperature of the AC. The decomposition of the AC yielded gas and formed initial bubbles. The initial bubbles were squeezed in the discharging region of a VPCU where the pressure is high, which caused the increasing pressure inside the bubbles. The bubbles drastically inflated and generated intense in-situ stretching force to act upon the melt when the materials flowed into the feeding region where the melt pressure is relatively low. The detailed process is shown in Figure 2b. The squeeze and rapid inflation process of the bubbles were repeated as the materials went through different VPCUs in the AC decomposition section, which realized the melt mixing process under the synergy of extensional deformation and ISBS. In this case, the CF agglomerates simultaneously received extensional deformation along the melt flow direction and the ISBS from the interior of the agglomerates, falling apart thoroughly and dispersing evenly in the matrix. Meanwhile, the heat generated by ISBS would increase the interface temperature between HDPE and UHMWPE to some extent and enhance the chain diffuse coefficient of UHMWPE. The main mechanism of this process can be depicted as follows: firstly, the synergy of intense stretching force from the bubbles and extensional deformation from VPCUs accelerated the dispersion of the dispersed phase in the matrix. Secondly, the higher temperature caused by ISBS promoted the molecular diffusion of UHMWPE, resulting in the better integration of HDPE/UHMWPE blend.

#### 2.2.4. Devolatilization and Metering 

The blends were forced into the devolatilization and metering section and further mixed and conveyed dominated by extensional deformation. The pressure of the melt in the exhaust unit drastically reduced due to the increased volume caused by the larger eccentric distance between the rotor and the stator of the exhaust unit. In this way, the originally compressed gas and the vaporized volatile matter were foamed in the melt simultaneously. The bubbles burst and the escaping gas was removed from the exhaust vent. Then the materials were metered and extruded from the outlet where different dies could be connected to extrude various types of products.

### 2.3. Materials

High density polyethylene pellets (HD7000F, melt flow index 0.04 g/10min, density 0.954 g/cm^3^) were supplied by PTT Public Co. Ltd., Bangkok, Thailand. 

UHMWPE resin (in powder form, molecular weight 2.6 × 10^6^ g/mol, mean particle size 190 μm) was provided by Shanghai Research Institute of Chemical Industry, Shanghai, China.

Short CFs (diameter 7–10 μm, length 6 mm, density 1.6–1.76 g/cm^3^) were supplied by Shanghai Lishuo composite technology Co. Ltd., Shanghai, China.

AC (C_2_H_4_N_4_O_2_, azodicarbonamide, decomposition temperature range 180–210 °C, amount of gas evolution 215–235 ml/g, purity greater than 99%) was produced from Jinan Hengrui Chemical Co., Ltd., Jinan, China.

### 2.4. Sample Preparation

The HDPE, UHMWPE and CFs were dried in a vacuum oven at 80 °C for 8 h. The temperature profile was 170, 175, 210, and 200 °C from hopper to die. The rotation rate was fixed at 30 rpm. Dried HDPE, UHMWPE, CFs, and AC were manually premixed by tumbling in a plastic zip-lock bag and subsequently fed into the VE for compounding. The total mass of HDPE/UHMWPE/CF blend with AC was 3000 g. The UHMWPE content was kept at 9 wt % with varied CF and AC loadings, the detailed designation and composition for HDPE/UHMWPE/CF composites can be found from the figures and tables in Section 3 below. In all cases, materials were cooled to room temperature in air and then granulated by a pelletizer after compounding. The pellets were dried again for further tests and characterization. Part of dried pellets were used directly for differential scanning calorimetry (DSC), TGA test, and some were compression mold with a compression molding machine (QLB-25/Q) and then machined into disks (Ø 25 × 1 mm) for rheological test. The molding temperature, molding time and molding pressure were 200 °C, 6 min, and 15 MPa, respectively. Some pellets were injection molded with an injection molding machine (DP-90) to prepare standard tensile test specimens (ASTM D638) and standard Notched izod test specimens (ASTM D256). The temperature profile was 190–200–210–210 °C. The injection pressure, holding pressure, and mold temperature were 60, 50 MPa, and 80 °C, respectively.

### 2.5. Characterization

#### 2.5.1. Morphology Observation

For morphology observation, the fractured standard Notched izod test specimens (The specimen dimensions were 80 mm × 10 mm × 4 mm) were used. The characterization of the morphology was carried out by using Scanning Electron Microscopy (SEM; Quanta 250, FEI, Hillsboro, OR, USA) at an accelerated voltage of 5 kV. The fractured surface of specimens was sputter coated with a thin gold layer to avoid charging during SEM imaging.

#### 2.5.2. Differential Scanning Calorimetry (DSC)

Thermal properties of HDPE/UHMWPE/CF composites were evaluated with a DSC204 differential scanning calorimeter(NETZSCH, Germany). The sample weighted about 5 mg was sealed in an aluminum pan and heated or cooled in a nitrogen atmosphere. At first, the samples were heated from room temperature to 180 °C at a rate of 10 °C/min to erase the thermal history and cooled at a rate of 10 °C/min to obtain the non-isothermal crystallization. Then the second heating run followed at a heating rate of 10 °C/min. 

#### 2.5.3. Thermogravimetric Analysis (TGA)

A TG209 Thermogravimetric Analyzer (NETZSCH, Germany) was used to analyze the thermal stability of HDPE/UHMWPE/CF composites. The weight of the samples was approximately 10 mg. The analyses were done from 30 to 600 °C at a heating rate of 10 °C/min under nitrogen flow (20 mL/min).

#### 2.5.4. Rheological Properties Analysis

Physica MCR302 rheometer (Anton Paar, Graz, Austria) equipped with a CTD620 convection oven was used to characterize the dynamic rheological behavior of the HDPE/UHMWPE/CF samples. The samples were tested at 190 °C with a scanning frequency range of 100–0.01 rad/s and strain amplitude of 1%. All tests were run under nitrogen purge at a flow rate of 5 mL/min.

#### 2.5.5. Mechanical Test

Tensile tests of HDPE/UHMWPE/CF composites were conducted by using an INSTRON universal machine (model 5566, Canton, MA, United States) with a tensile speed of 20 mm/min according to the GB/T 1447-2005 standard. The notched impact properties were examined according to the standard of GB/T 8814-1998 with a pendulum impact tester (POE2000, Instron, Norwood, MA, USA).

All tests were performed at ambient temperature (25 °C), and five specimens were used in each test (X_i_) to obtain the average value ($\bar{X}$). The standard deviation (E_X_) was obtained by Equation (1):

(1) $$E_{X}=\sqrt{\frac{1}{n-1}\overset{n}{\underset{i=1}{\sum }}{\left(X_{i}-\bar{X}\right)}^{2}}$$

## 3. Results and Discussion

### 3.1. Morphology Observation

**Figure 3:**
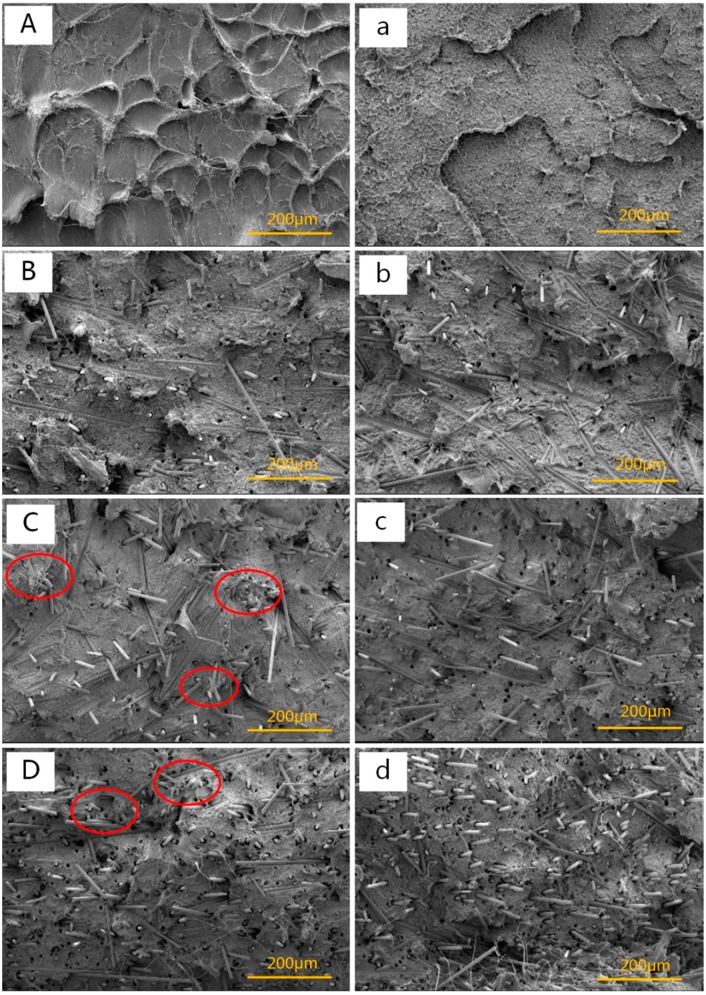
SEM micrographs (500×) of the fractured surfaces for the composites containing (**A**,**a**) 0, (**B**,**b**) 4, (**C**,**c**) 8, and (**D**,**d**) 12 wt % CFs.

Figure 3 provided the SEM images of the fractured impact surface for HDPE/UHMWPE/CF composites under different CF loadings with (right column) and without (left column) adding AC, respectively. The UHMWPE and AC content were fixed at 9 and 0.4 wt %, respectively. Comparing Figure 3a with Figure 3A, it can be found that the fractured surface was smoother and there was no distinct phase interface between disperse phase (UHMWPE) (raised borders) and the HDPE matrix when AC added in the composites. This is due to the fact that UHMWPE dispersed evenly and there was a fine integration of HDPE/UHMWPE blend under the synergy of extensional deformation and ISBS which reduced the amount of stress concentration points caused by the UHMWPE agglomerates. As shown in Figure 3B–D, there was no CF aggregates in the composites when the CF loading is low (Figure 3B), however, a few CF aggregates emerged in the composites when the CF loading increased (Figure 3C,D). In comparison, it could be noticed that CFs had a better dispersion after adding AC (Figure 3b–d). The better dispersion of CFs can be ascribed to the following two reasons: firstly, early bubbles were inflating rapidly into swollen bubbles among the CF aggregates in diverging region during ISBS, giving rise to intense bubble stretching force inside the CF aggregates which suffered pulsatile extensional deformation along the direction of melt flow. Under the synergy of these two forces, the CF aggregates were prone to fall apart thoroughly and disperse evenly in the matrix (as illustrated in Figure 2b). Secondly, the melt pressure and temperature increased with adding AC, which can accelerate the diffusivity of CFs in the blends [37], leading CFs to dispersing in the matrix more easily. Comparing Figure 3d with Figure 3D, there were more highly and regularly oriented fibers. This was attributed to better dispersion of CFs under the synergetic action of extensional deformation and ISBS, causing CFs to be highly oriented along the flow direction (vertical to the fractured surface) during the injection molding process for making the impact test specimens. Meanwhile, we could find a number of pullout traces of fibers and micro voids in the matrix, indicating interfacial bonding between matrix and some fibers was weaker than the strength of the fibers. Thus, the fibers tended to be pulled out of the matrix rather than breaking during impact.

**Figure 4:**
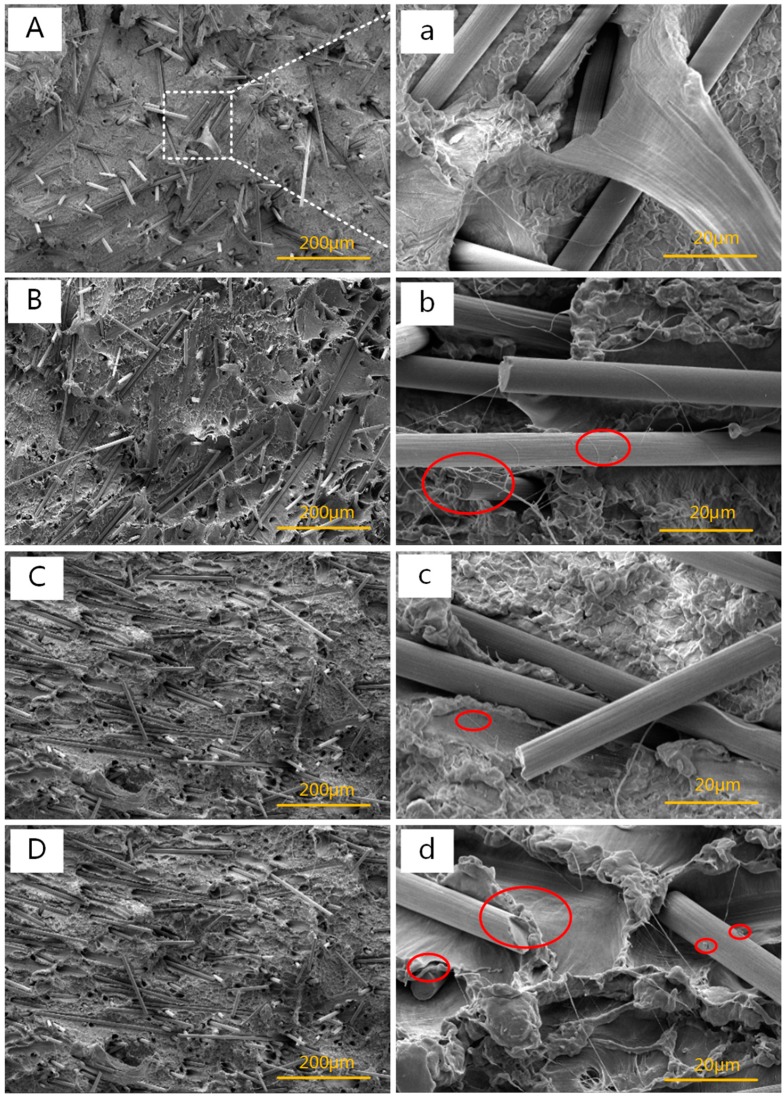
SEM micrographs (500×/5000×) of the fractured surfaces for the composites with different AC contents. (**A**,**a**) 0, (**B**,**b**) 0.2 wt %, (**C**,**c**) 0.4 wt %, and (**D**,**d**) 0.6 wt %.

Figure 4 shows the SEM images of HDPE/UHMWPE/CF composites with different AC contents. The UHMWPE and CF contents were fixed at 9 and 8 wt %, respectively. As shown in Figure 4A–D, the CFs’ orientation was getting more and more obvious and the fractured impact surface showed more and more traces of ductile fracture with increasing AC content. As clearly seen in high magnification images (Figure 4a–d), the “wire drawing” behavior of UHMWPE under higher content AC (Figure 4b–d) was more pronounced than that under lower content AC (Figure 4a), and the UHMWPE filaments were turning thinner and thinner with the increasing AC content. The phenomena are due to the evenly-dispersed CFs and the disentanglement of the long UHMWPE chains under more powerful in-situ bubble stretching force synergized with periodically changed extensional deformation with the increased AC content. Meanwhile, we could find that the “wire drawing” behavior of UHMWPE happened near CFs which was getting closely attached to the matrix with increasing AC content. Particularly, there were a few polymer resins left on the surface of fibers (marked in Figure 4b,d). These can be related to the following facts: first, the better-dispersed CFs under the synergy of extensional deformation and more powerful ISBS have greater adhesion stress with the matrix. Second, the macromolecular chains (the part with high molecular weight) of UHMWPE will selectively adsorb on the surface of the CFs [38]. Third, some bubbles during ISBS tend to selectively expand among the CF aggregations [39]. Moreover, the heat generated during ISBS will increase the melt temperature to some extent, enhancing the chain diffuse coefficient of UHMWPE. Eventually, a better integration of HDPE/UHMWPE/CF composite might be obtained.

### 3.2. Thermogravimetric Analysis

**Figure 5:**
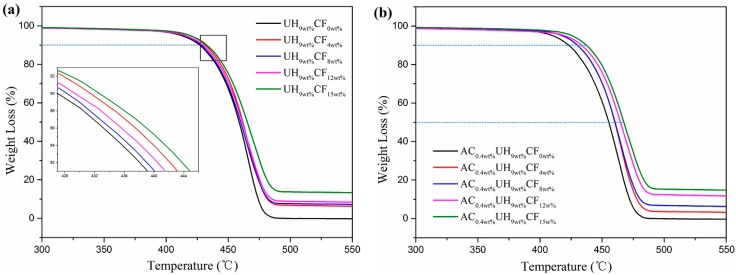
The TGA curves of the composites under different CF loadings (**a**) without AC (**b**) with 0.4 wt % AC.

Thermal stability of CF-reinforced composites for various applications is necessary in determining their end use. The TGA curves of HDPE/UHMWPE and HDPE/UHMWPE/CF composites under different CF loadings with (right) and without (left) AC were shown in Figure 5. Thermal stability parameters such as the temperature at 10 wt % weight loss (*T*_10%_), the temperature at 50 wt % weight loss (*T*_50%_), the maximum decomposition temperature (*T*_max_) and the char residue at 597.7 °C (Cr) were collected in Table 1. It could be observed from Figure 5 and Table 1 that the addition of CFs resulted in an increase in thermal stability of the composites as *T*_10%_, *T*_50%_, and *T*_max_ all shifted to a higher temperature with increasing CF loading. This could be interpreted as follows: first, the heat absorption capacity of CF is higher than that of HDPE and UHMWPE. As CF loading increased, the fibers in the composites absorbed more heat, therefore, higher temperature was required to achieve the threshold energy for commencement of the degradation process. Similarly, another research found the introduction of inorganic fillers into polymer resulted in improvement in thermal stability of polymers [40]; second, the CFs could form a barrier that obstructed the nitrogen diffusion, thus retarding the degradation of HDPE and UHMWPE; third, the CFs could establish some interaction with HDPE matrix and formed a network’s structure which was responsible for mobility restriction of HDPE chains [41]. Meanwhile, we could find the addition of AC led to a decrease in thermal stability of the HDPE/UHMWPE composite. However, the addition of AC gave rise to the enhancement in *T*_10%_, *T*_50%_, and *T*_max_ under pretty high CF loading (over 8 wt %). Especially, *T*_10%_ and *T*_max_ increased by 4.5 and 2.2 °C respectively when CF loading was 12 wt %. These could be the comprehensive results caused by the following facts: on the one hand, the bubbles expansion generated during ISBS produces a tensile rate of up to 10^6^ s^−1^, and the bubbles vibration frequency reaches the ultrasonic range [31,42], which can promote the CFs to disperse better in the matrix and the separated fibers have much greater specific surface area that strengthened the adhesion stress between CF and the matrix, improving thermal stability of the composites; On the other hand, the high tensile rate and vibration frequency of the bubble will inevitably cause the polymer molecular chain to break [43], reducing the thermal stability of the composites. Meanwhile, as discussed in Section 3.1, when CF loading was low, the CFs could be well-dispersed enough in the HDPE without adding AC (Figure 3B). Hence, the addition of AC had little impact on dispersion of the CFs but greatly accelerated the breakage of the matrix molecular chain. However, when CF loading was high, the CFs dispersed more evenly in the matrix under the synergistic effect of extensional deformation and ISBS (Figure 3c,d). In this case, the synergistic action had a better effect on CFs’ dispersion than on the fracture of the matrix. Most noteworthy, when CF loading was 15 wt %, the *T*_10%_ of the composite with 0.4 wt % AC increased by 14.2 °C compared to that without CF loading.

**Figure 6:**
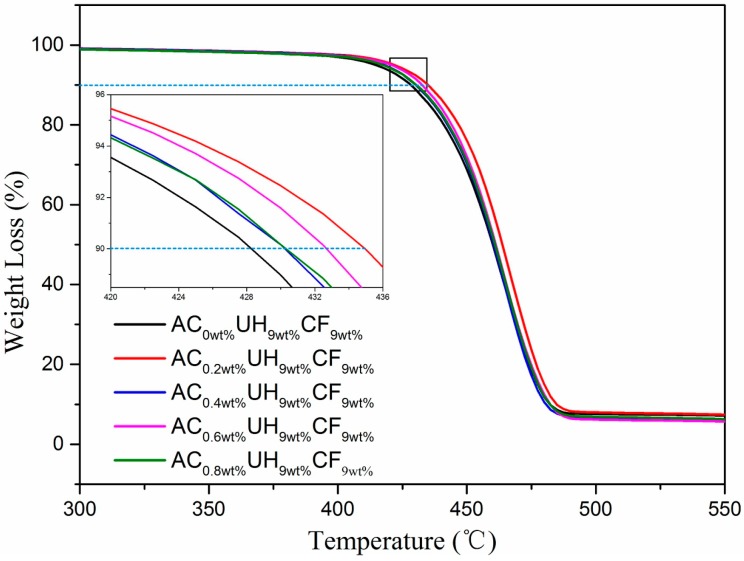
The TGA curves of HDPE/UHMWPE/CF composites with different AC contents.

The TGA curves and corresponding thermal stability parameters of HDPE/UHMWPE/CF composites under different AC contents were shown in Figure 6 and Table 2. As shown in Figure 6 and Table 2, the thermal stability of AC_0.2wt%_UH_9wt%_CF_9wt%_ was optimal. Compared with AC_0wt%_UH_9wt%_CF_9wt%_, the *T*_10%_, *T*_50%_, and *T*_max_ increased by 6.3, 2.4, and 1.9 °C, respectively. As AC content kept on increasing, the thermal stability of the composites decreased but was still higher than that prepared without AC. As illustrated above, the uniformly-dispersed CFs under the synergy of extensional deformation and ISBS formed a network’s structure which worked as mobility restriction of HDPE chains, resulting in an increase in thermal stability of the composites. However, as AC content continued increasing, the molecular chain breakage of the matrix was getting more and more severe, leading to a decrease in thermal stability. In addition, the char residues at 597.7 °C (C_r_) in Table 1 and Table 2 were CFs. They were lower than the designed CF loading. This is because that CFs were fibrous fillers and easy to be flocculated to block the feed port during feeding, which hindered some CFs in entering the vane extruder. However, the reduction of the char residues does not affect the experimental analysis results.

### 3.3. DSC Analysis

**Figure 7:**
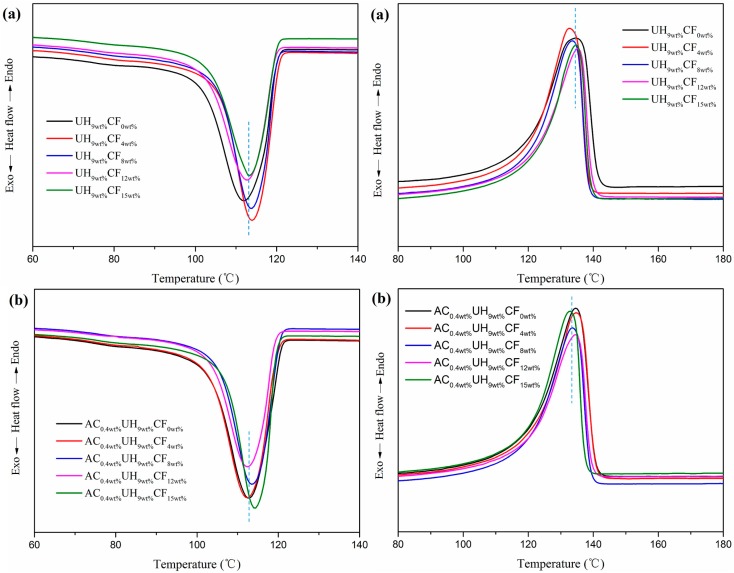
Effect of CF loadings on HDPE/UHMWPE/CF composites’ melting curves (**a**) without AC, (**b**) with AC.

The effect of CF loading on composites’ thermal properties is shown in Figure 7. Results of the DSC analysis were summarized in Table 3, in which the crystallization temperature (*T*_c_), melting temperature (*T*_m_), melting enthalpy (Δ*H*_m_), and the percentage crystallinity (*X*_c_) of HDPE/UHMWPE and HDPE/UHMWPE/CF composites were collected. The *X*_c_ was calculated according to Equation (2) [44]:

(2) $$X_{c}=\frac{\Delta H_{m}}{f_{HDPE}\times \Delta H_{m}^{0}}$$

where *f_HDPE_* is the HDPE mass fraction in the composites, and $\;\Delta H_{m}^{0}$ is the melting enthalpy of 100% crystalline polyethylene which is equal to 287 J/g [45].

As seen from Figure 7 and Table 3, the heating and cooling curves of the HDPE/UHMWPE/CF composites showed only one peak, and the melting range of the composites did not change significantly, indicating that the crystal formed by HDPE matrix and UHMWPE in the blend was single crystal or eutectic, which was consistent with the findings by Chen [21] and Kyu [46]. It had previously been reported that the poor mixing of UHMWPE/LDPE blend could cause dual exothermic peaks in the DSC curve [47]. Hence, the HDPE and UHMWPE were well mixed by VE, and the addition of CFs did not affect the co-crystallization behavior of HDPE/UHMWPE blend. When there was no AC added, the *T*c of the HDPE/UHMWPE/CF composites increased significantly and the crystallization peak became narrower with the addition of CFs. It implied that CFs acted as heterogeneous nucleation in the HDPE matrix and induced crystallization. When CF loading was 4 wt %, the *T*c and *X*_c_ were the highest among HDPE/UHMWPE/CF composites without adding AC, denoting that the CFs were well-dispersed in the matrix, which was consistent with the results of the morphology observation (Figure 3B). However, neither AC nor the high loading CFs (over 8 wt %) enhanced but decreased the *X*_c_ of HDPE/UHMWPE/CF composites. Compared with the composites without AC, the *X*_c_ of the composites with AC decreased by 2%–6% (Table 3). The phenomenon was the competitive result of the factors as follows: as for CFs, on the one hand, the dispersed CFs are beneficial to HDPE crystallization. On the other hand, the CF agglomerates act as restriction sites for the matrix, which obstructs them from obtaining a highly ordered spherulite structure and decreases the crystal growth in the matrix [48,49]. The higher the CF loading was, the more agglomeration phenomena there would be (Figure 3C,D); as for AC, although AC could further improve the dispersion of the CFs, the high tensile rate and vibration frequency of the bubble generated during ISBS will inevitably cause the matrix molecular chain to break and degrade which could not crystallize when the temperature was cooled down and in turn decreased the *X*_c_ to some degree.

As we all know, the CFs dispersed in the HDPE matrix could shield the conduction of heat to crystallites until at higher temperatures that the heat flow is sufficient to melt down the crystallites [50]. However, from Figure 7 and Table 3, we can find that the *T*_m_ of the composites without adding AC did not have visible change with the increasing CF loading. There are two possible factors which cause this phenomenon: on the one hand, the CFs could induce the crystallization of the matrix and shield conduction of heat to crystallites, which would have resulted in increasing of the *T*_m_. On the other hand, the agglomerates of the fibers prevented macromolecular segments from obtaining requisite alignment of crystal lattices under pretty high CF loading (over 8 wt %), which hindered the crystallization and decreased the *X*_c_, and the *T*_m_ would have decreased with the lower value of *X*_c_. Furthermore, the addition of AC did not have much effect on the *T*_m_.

**Figure 8:**
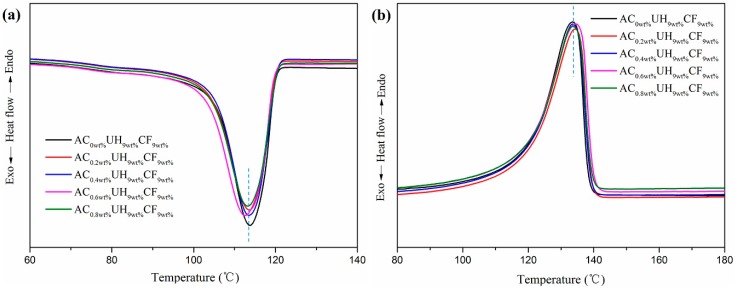
Effect of AC contents on HDPE/UHMWPE/CF composites’ melting curves (**a**) cooling, (**b**) second heating.

From Figure 8 and Table 4, it could be seen that the *T*_c_ and *X*c of HDPE/UHMWPE/CF composites gradually decreased with increasing AC content. The result was ascribed to these aspects: one the one hand, the fracture and decomposition of the matrix molecular chain were accelerated with increasing AC content. One the other hand, UHMWPE worked as heterogeneous nucleation sites to induce crystallization of the matrix and it was sensitive to the high tensile rate and vibration frequency under ISBS. As the AC content increased, more and more UHMWPE molecular chain got fractured and decomposed. Moreover, the *T*_m_ of the composites was almost independent of AC content.

### 3.4. Rheological Properties Analysis

**Figure 9:**
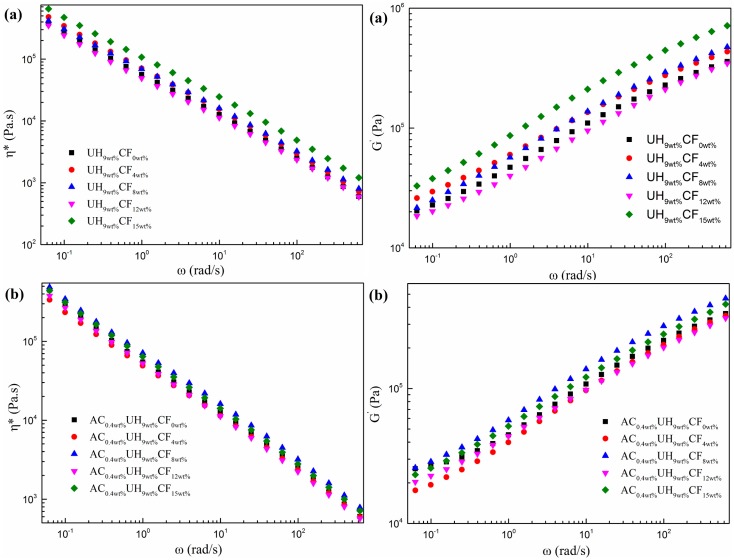
Complex viscosity and storage modules of the composites prepared under different CF loadings (**a**) without AC, (**b**) with AC.

The variation of complex viscosity and storage modulus for the HDPE/UHMWPE/CF composites prepared without (Figure 9a) and with AC (Figure 9b) along frequency under different CF loadings were demonstrated, respectively. As seen in Figure 9a, the complex viscosity of the composites decreased as function of frequency, exhibiting a very strong shear thinning effect. As the CF loading increased, the complex viscosity and storage modulus of the composites without AC increased significantly over the entire frequency range. This is believed to have resulted from the facts below: on the one hand, the molecular chain of HDPE was less entangled and the CFs formed a physical network in the matrix when CF loading was 15 wt%, which is defined as solid-like behavior [51]. On the other hand, the increase of storage modulus was mainly determined by the stress transfer between the matrix and the fibers [52]. Moreover, the interaction between fiber–fiber and fiber–polymer molecular restricted the movement of the polymer molecular chains [53]. Figure 9b shows the influence of CF loading on the complex viscosity and storage modulus of the blends. Compared with Figure 9a, the complex viscosity and storage modulus of the composites prepared with AC decreased to some degree. It is strange that when 4 wt % CF added, the complex viscosity and storage modulus of the composite were lower than these without adding CFs. The results can be explained as follows: firstly, the CFs which have strong interaction with UHMWPE molecular chains (discussed in Section 3.1) dispersed evenly in the matrix under 4 wt % CFs (Figure 3b) and the bubbles generated during ISBS tend to selectively expand among the CFs where UHMWPE molecular chains accumulated on, promoting the disentangling of the UHMWPE molecular chains [54]; secondly, as mentioned in Section 3.2, the high tensile rate and vibration frequency during ISBS will lead to the breakage and degradation of molecular chain of HDPE and UHMWPE. The degradation by-product of the HDPE and UHMWPE played as plasticizing agent during rheological test.

**Figure 10:**
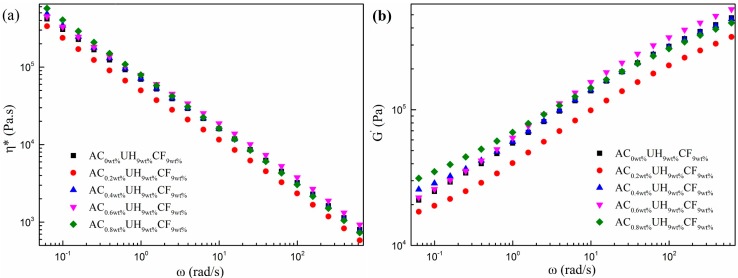
Complex viscosity and storage modules of the composites prepared under different AC contents (**a**) complex viscosity, (**b**) storage modules.

The way in which the complex viscosity (left) and storage modulus (right) affected by AC content is shown in Figure 10. As shown in Figure 10b, the slope of curve in low-frequency region became smaller with the increasing AC loading, and the storage modulus of the composites was obviously increased when AC was added. As we all know, the rheological behavior under low frequency region reflects the dispersion state of the fillers in the polymer matrix. From Figure 4, we know that the CFs dispersed more evenly in the matrix with increasing AC content. The better-dispersed CFs promoted by ISBS could further restrict the motion of polymer chains and thus gave rise to the increase in storage modulus. From Figure 10a, we can find the complex viscosity was slightly increased with the addition of AC. As stated in SEM results, this is due to the better dispersion of the CFs under the synergy of extensional deformation and ISBS.

### 3.5. Mechanical Properties

#### 3.5.1. Tensile Strength

**Figure 11:**
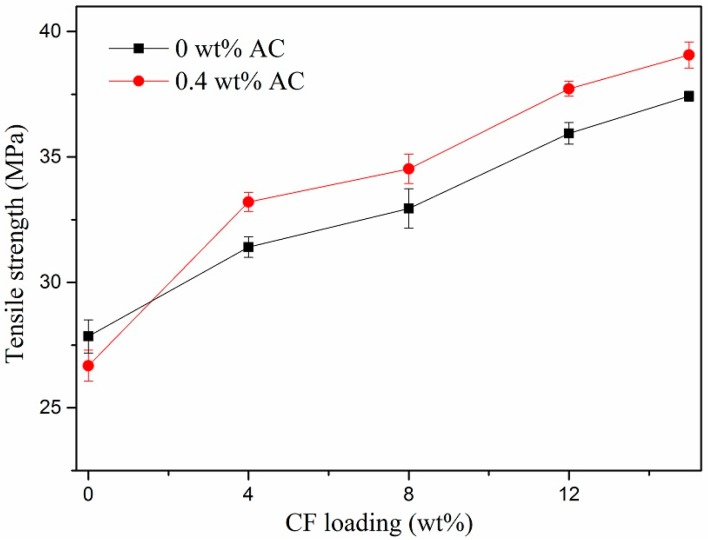
Effect of CF loadings on tensile strength of the composites.

Figure 11 illustrats the effect of CF loadings on tensile strength of HDPE/UHMWPE/CF composites prepared without and with AC respectively. The UHMWPE content was kept at 9 wt %. As illustrated in Figure 3C,D, there was an obvious aggregation phenomenon in the matrix, resulting in stress concentration that caused the decrease of the tensile strength of the composites. As a result, the tensile strength should have decreased. However, the tensile strength of the composites prepared without AC increased (from 28 to 37.4 MPa) as CF loading increased from 0 to 15 wt %. Meanwhile, the tensile strength increased by 4.5% when 0.4 wt% AC was added. There are two possible reasons which caused these results. On the one hand, the CFs with high concentration could shield the conduction of heat to the matrix, which reduced the degradation of polymer molecular chains. On the other hand, the CFs under the synergy of ISBS and extensional deformation were better-dispersed and highly-oriented in the matrix (Figure 3), and the separated CFs had greater specific surface area and more sufficient adhesion stress with matrix, which will be favorable for stress transfer at the interface when external forces are exerted on the composites. However, when there was no CF added, the tensile strength of HDPE/UHMWPE composite decreased from 27.1 to 26.5 MPa after adding 0.4 wt % AC. This phenomenon can be ascribed to the degradation of the UHMWPE and HDPE chains accelerated by ISBS, which lead to reduction in tensile strength. 

**Figure 12:**
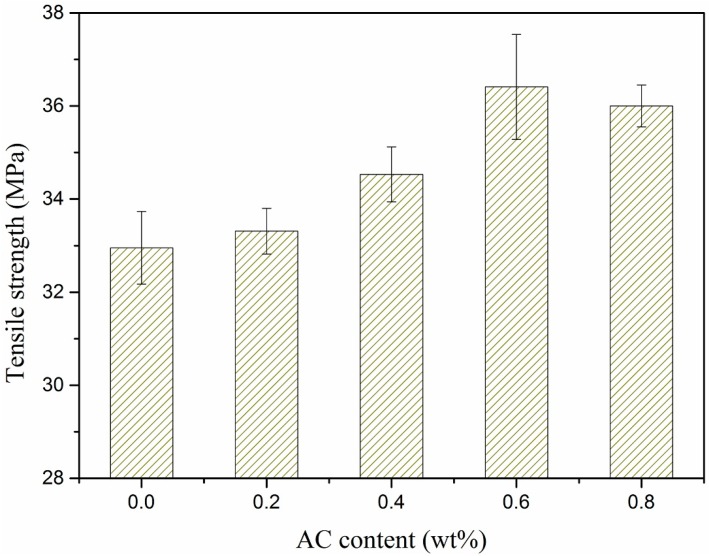
Effect of AC contents on tensile strength of the composites.

Effects of the AC contents on tensile strength of the HDPE/UHMWPE/CF composites were investigated (Figure 12). The contents of CF and UHMWPE were both maintained at 9 wt %. The tensile strength increased fast at first and then leveled off with the increasing AC content. Tensile strength of the composite increased by 10% when 0.6 wt % AC was added. Most noteworthy, compared with HDPE/UHMWPE composite without AC, tensile strength of HDPE/UHMWPE/CF composite increased by 30% when 0.6 wt % AC and 9 wt % CF were added. The improvement of the tensile strength is ascribed to the homogeneous distribution of the CFs and UHMWPE in the matrix and the close attachment between the CFs and the matrix (Figure 4) which was discussed in Section 3.1. and the decrease of the tensile strength is not only due to the thermo–mechanical degradation of the HDPE and UHMWPE chains, but also the breakage of CFs caused by high tensile rate and vibration frequency during ISBS.

#### 3.5.2. Impact Strength

**Figure 13:**
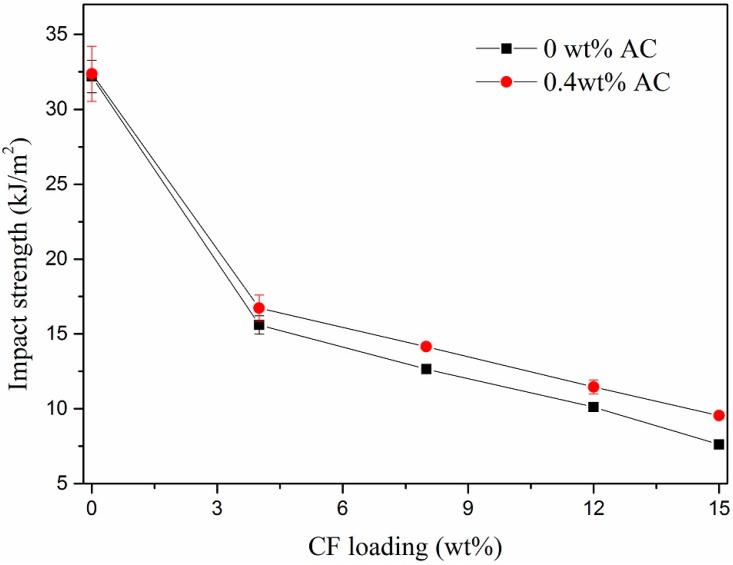
Effect of CF loadings on impact strength of the composites.

Figure 13 illustrats the effect of CF loadings on impact strength of HDPE/UHMWPE/CF composites prepared with and without AC respectively. The UHMWPE content was fixed at 9 wt %. The impact strength decreased with increasing CF loading, however, it increased to some extent when 0.4 wt % AC added under the same CF loading. This can be explained as below: first, the addition of CFs would affect the integrity of the HDPE matrix and worsen the ability of matrix molecular chains to withstand plastic deformation, reducing the materials’ impact strength. Second, after the synergy of ISBS and extensional deformation, the debonding of CFs with better dispersion from the matrix induced more shear yielding of the matrix which resulted in extra dissipation of impact energy.

**Figure 14:**
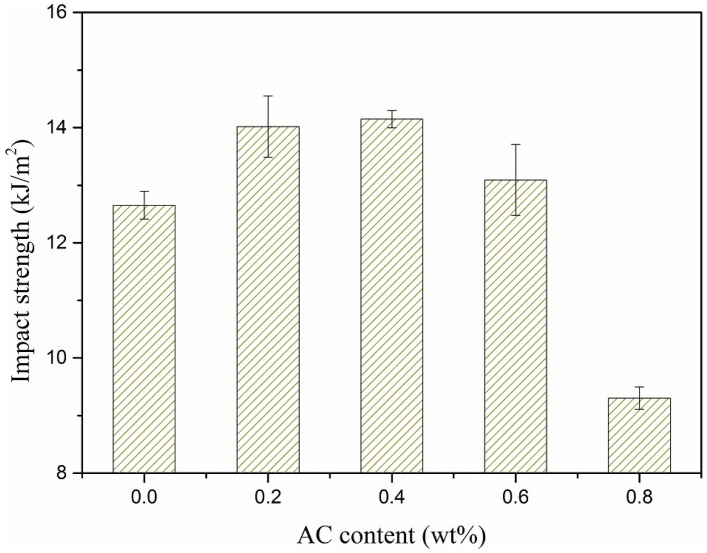
Effect of AC contents on impact strength of the composites.

Figure 14 shows the effect of AC content on the HDPE/UHMWPE/CF composites’ impact strength. The contents of CF and UHMWPE were both kept at 9 wt %. As discussed above, the addition of AC (not exceeding 0.4%) improved the impact strength. However, when AC content was more than 0.4 wt %, the impact strength turned to decrease, especially, there was a sharply decline when 0.8 wt % AC added. As mentioned in Section 3.2, the synergy of extensional deformation and ISBS could promote the dispersion of CF in the matrix but accelerate the degradation of matrix molecular chain as well. When AC content was relatively low, the dispersion effect dominated, resulting in an increase in impact strength of the composites; when the AC content was approaching or exceeding 0.6%, the degradation effect dominated, causing a sharp decrease in impact strength.

## 4. Conclusions

A novel melt extrusion method and corresponding apparatus under the synergy of extensional deformation and ISBS was put forward. The novel extrusion method makes AC disperse evenly in the melt first under extensional deformation and then decompose, expand to bring about the synergy of extensional deformation and ISBS. HDPE/UHMWPE/CF composites with fine dispersion of CFs and UHMWPE in matrix were obtained. The crystallinity and crystallization rate increased due to the better dispersion of the CFs in the matrix which acted as the nucleating point at certain CF and AC content. The addition of CF and AC could lead to a marked increase in tensile strength. We can conclude that the synergy of extensional deformation and ISBS is a green way for compounding the HDPE/UHMWPE/CF composites with high AC utilization rate.

## Figures and Tables

**Table 1:** TGA data for the composites under different CF loadings with and without AC.

Samples	*T*_10%_ (°C)	*T*_50%_ (°C)	*T*_max_ (°C)	*C_r_ (*%*)*
UH_9wt%_CF_0wt%_	425.5	459.1	475.6	0.35
AC_0.4wt%_UH_9wt%_CF_0wt%_	423.2	455.8	475.9	0.43
UH_9wt%_CF_4wt%_	431.2	460.5	479.0	2.52
AC_0.4wt%_UH_9wt%_CF_4wt%_	430.2	459.8	478.3	2.88
UH_9wt%_CF_8wt%_	428.6	460.9	480.3	6.70
AC_0.4wt%_UH_9wt%_CF_8wt%_	430.1	459.2	478.2	6.57
UH_9wt%_CF_12wt%_	429.4	461.6	480.7	10.99
AC_0.4wt%_UH_9wt%_CF_12w%_	433.9	462.2	482.9	10.96
UH_9wt%_CF_15wt%_	432.5	465.0	484.1	14.79
AC_0.4wt%_UH_9wt%_CF_15w%_	437.4	464.0	484.2	14.19

**Table 2:** TGA data for high density polyethylene (HDPE)/ ultrahigh molecular weight polyethylene (UHMWPE)/CF composites with different AC contents.

Samples	*T*_10%_ (°C)	*T*_50%_ (°C)	*T*_max_ (°C)	*C_r_ (*%*)*
AC_0wt%_UH_9wt%_CF_9wt%_	428.6	459.9	480.3	6.70
AC_0.2wt%_UH_9wt%_CF_9wt%_	434.9	462.3	482.2	6.12
AC_0.4wt%_UH_9wt%_CF_9wt%_	430.1	459.2	478.2	6.57
AC_0.6wt%_UH_9wt%_CF_9wt%_	432.6	460.1	480.8	6.17
AC_0.8wt%_UH_9wt%_CF_9wt%_	430.5	460.6	480.6	6.64

**Table 3:** Differential scanning calorimetry (DSC) parameters of melting and crystallization for samples under different CF loadings (Second heating cycle).

Samples	*T*_c_ (°C)	*T*_m_ (°C)	Δ*H*_m_ (J/g)	*X*c (%)
UH_9wt%_CF_0wt%_	112.0	134.7	176.1	60.1
AC_0.4wt%_UH_9wt%_CF_0wt%_	112.9	134.6	165.4	56.4
UH_9wt%_CF_4wt%_	113.9	132.7	174.9	62.2
AC_0.4wt%_UH_9wt%_CF_4wt%_	112.3	134.8	158.6	56.3
UH_9wt%_CF_8wt%_	113.8	133.7	155.6	57.7
AC_0.4wt%_UH_9wt%_CF_8wt%_	113.5	133.6	150.3	55.7
UH_9wt%_CF_12wt%_	112.7	135.4	154.1	59.7
AC_0.4wt%_UH_9wt%_CF_12wt%_	112.4	134.6	141.9	55.0
UH_9wt%_CF_15wt%_	113.2	134.6	152.1	60.0
AC_0.4wt%_UH_9wt%_CF_15wt%_	114.3	132.9	137.3	55.1

**Table 4:** DSC parameters of melting and crystallization for samples with different AC contents (Second heating cycle).

Samples	*T*_c_ (°C)	*T*_m_ (°C)	Δ*H*_m_ (J/g)	*X*c (%)
AC_0wt%_UH_9wt%_CF_9wt%_	113.8	133.7	155.6	57.7
AC_0.2wt%_UH_9wt%_CF_9wt%_	113.6	134.4	151.1	56.6
AC_0.4wt%_UH_9wt%_CF_9wt%_	113.5	133.6	150.3	56.4
AC_0.6wt%_UH_9wt%_CF_9wt%_	112.6	134.4	150.0	56.2
AC_0.8wt%_UH_9wt%_CF_9wt%_	113.2	133.6	150.6	56.5
